# A Novel Densovirus Isolated From the Asian Tiger Mosquito Displays Varied Pathogenicity Depending on Its Host Species

**DOI:** 10.3389/fmicb.2019.01549

**Published:** 2019-07-05

**Authors:** Jing Li, Yunqiao Dong, Yan Sun, Zetian Lai, Yijie Zhao, Peiwen Liu, Yonghui Gao, Xiaoguang Chen, Jinbao Gu

**Affiliations:** ^1^Guangdong Provincial Key Laboratory of Tropical Disease Research, Department of Pathogen Biology, School of Public Health, Southern Medical University, Guangzhou, China; ^2^Reproductive Medical Centre of Guangdong Women and Children’s Hospital, Guangzhou, China

**Keywords:** mosquito-borne viral diseases, mosquito densoviruses, vector control, *Aedes albopictus*, mosquito entomopathogenic viruses

## Abstract

Mosquito-borne viral diseases (MBVDs) continue to pose a significant global public health burden. Mosquito control remains a core intervention strategy in integrated mosquito management (IMM) programs to reduce the transmission of MBVDs. Mosquito densoviruses (MDVs) are mosquito-specific entomopathogenic viruses, and their attractive biological and pathogenic characteristics make MDVs potential biological control agents as alternatives to traditional chemical pesticides. However, different viral strains vary greatly in their pathogenicity against different mosquito species, which poses an obstacle for the wide application of MDVs in vector control. In this study, a novel MDV, Aedes albopictus densovirus-7 (AalDV-7), was isolated from field-collected *Aedes albopictus* in the dengue-endemic area of Guangzhou, China. The complete 4,048 nt genome of AalDV-7 was cloned and sequenced, and the transcription and translation of three open reading frames (ORFs) were characterized. Phylogenetic analysis indicated that AalDV-7 clustered with MDVs mostly isolated from indigenous mosquitoes. The pathogenicity of AalDV-7 to *A. albopictus*, *Aedes aegypti*, and *Culex quinquefasciatus* larvae was completely different, and the median lethal dose (LD_50_) of AalDV-7 in *A. albopictus* which was 10^9.48^ genome equivalents per ml (geq/ml) was 12 and 46 times lower than those in *A. aegypti* (10^10.56^ geq/ml) and *C. quinquefasciatus* (10^11.15^ geq/ml). Furthermore, the median lethal time (LT_50_) value in *A. albopictus* (7.72 days) was 25% and 26% shorter than those in *A. aegypti* (10.24 days) and *C. quinquefasciatus* (10.42 days) at a titer of 10^11^ geq/ml. Furthermore, the mortality of AalDV-7-infected mosquitoes increased in a dose-dependent manner, and the highest mortality was found in *A. albopictus* larvae exposed to 10^11^ geq/ml AalDV-7 (82.00%). Sublethal effects analysis also showed that AalDV-7 infection significantly decreased pupation and emergence rates. The 1st–2nd instar larvae of all three mosquito species showed a near 100% infection rate, and the highest relative vial titer (305.97 ± 67.57 geq/ng) was observed in the 1st–2nd instar larvae of *C. quinquefasciatus*. These pathogenic characteristics make AalDV-7 a potential MBVDs control agent in China, whereas its negligible pathogenicity and high infection rate and viral dose *in vivo* make it a good candidate for gene delivery vectors in *C. quinquefasciatus* gene function analysis. In conclusion, the continuous discovery and isolation of new MDVs enrich the pool of mosquito entomopathogenic viruses and provide a variety of choices for optimal MDVs or combinations of MDVs to target certain mosquitoes.

## Introduction

Mosquito-borne viral diseases (MBVDs) pose a serious threat to global public health. Dengue fever is the most important arboviral disease in tropical and subtropical areas and is especially prevalent in Southeast *Asia*, regions in the West Pacific Ocean, and Southern Africa (Radke et al., 2012; Schaffner and Mathis, 2014). This disease is caused by dengue virus transmitted by *Aedes* genus mosquitoes, mainly *Aedes aegypti* and *Aedes albopictus* (Asian tiger mosquito) (Knudsen, 1995), which are regarded as the primary dengue virus vector in mainland China and contribute to large dengue outbreaks with serious consequences, especially the 2014 outbreak in Guangzhou (Zhao et al., 2016; Luo et al., 2017), which was the worst in two decades (Wang et al., 2019). Furthermore, *A. albopictus*, which is one of world’s worst invasive alien species, exhibits great adaptability to diverse ecological conditions, and its geographic distribution is expanding through tropical and temperate regions (Mariangela et al., 2013). Currently, no specific antiviral therapy or vaccines are available against dengue. Thus, mosquito control has been the principal tool for dengue prevention. However, the traditional method of chemical pesticide application has generated complex problems because of the high level of toxicity to the environment, the consequent safety risks for humans, and insecticide resistance in mosquitoes, and new approaches to combat this disease are needed (Turner et al., 2016; Auteri et al., 2018). Moreover, dengue virus must live in the mosquito for some time during its life cycle before its transmission to humans. Therefore, in addition to controlling the population of mosquitoes, the inhibition of dengue virus reproduction in mosquitoes could also effectively minimize the prevalence of diseases caused by dengue virus.

Mosquito densoviruses (MDVs) are autonomous, non-enveloped paraspherical DNA viruses, 20–25 nm in diameter with icosahedral symmetry that contain a single-stranded linear DNA genome, and the majority of MDVs belong to the *Brevidensovirus* genus in the subfamily *Densovirinae* within the *Parvoviridae* family (Cotmore et al., 2014). In 1972, the first MDV, Aedes aegypti densovirus (AeDV), was discovered in an *A. aegypti* laboratory colony (Lebedeva et al., 1972), and MDVs have been successively isolated from laboratory strains and natural populations of mosquitos such as *A. aegypti* (Kittayapong et al., 1999; Sivaram et al., 2009), *A. albopictus* (Boublik et al., 1994; Fu et al., 2017), *Culex pipiens* (Jousset et al., 2000), *Toxorhynchites splendens* (Pattanakitsakul et al., 2007), *Anopheles gambiae* (Ren et al., 2008), *Anopheles minimus* (Rwegoshora et al., 2000), and other mosquito cell lines over the past several decades (Jousset et al., 1993; O’Neill et al., 1995; Mosimann et al., 2011).

Nearly all MDVs exert detectable pathological effects on mosquitoes, although different viral strains vary greatly in their pathogenicity to different mosquito species (Ward et al., 2001; Ledermann et al., 2004; Rwegoshora and Kittayapong, 2004; Carlson et al., 2006; Hirunkanokpun et al., 2008; Ren et al., 2014; Suzuki et al., 2015). MDVs can disseminate to multiple tissues and organs of larvae including muscle, the fatbody, the midgut, salivary glands, and the malpighian tubules (Afanasiev et al., 1999; Ward et al., 2001). Infected larvae exhibited MDV-associated pathogenic characteristics including decreased mobility, a deformed and distended body, and the loss of the pigment, and severe infection resulted in the mortality of the larvae and pupae (Buchatsky, 1989; Barreau et al., 1996; Jousset et al., 2000). Moreover, low-dose MDV infection caused sublethal effects that were apparently restricted to mosquitoes, and MDVs were relatively stable in aqueous environments and spread and persisted in mosquito populations (Ledermann et al., 2004; Carlson et al., 2006). Furthermore, their pathogenicity can be significantly improved by genetic engineering techniques, such as the use of vectors to express insect-specific toxins, specific shRNAs or artificial miRNAs that target essential genes involved in the development, growth or physiology of mosquitoes (Gu et al., 2010, 2011; Liu et al., 2016). Thus, with their attractive biological characteristics, MDVs show potential for use in biological mosquito control.

Despite their promising application prospects, the unpredictable pathogenicity of MDVs is an obstacle for their further development and application. In general, the mosquito mortality rate shift from 10% to more than 90% which depended on the viral strain and species of mosquito even between geographical strains (Hirunkanokpun et al., 2008). Furthermore, Anopheles gambiae densovirus (AgDV), which was originally isolated from *An. gambiae* Sua5B cells (Ren et al., 2008), displayed negligible pathogenicity to its host, *An. gambiae*, even when the larvae were exposed to approximately 3 × 10^11^ viral genome equivalents per ml (geq/ml) (Ren et al., 2014). Therefore, the discovery and isolation of these new MDVs enriches the pool of mosquito entomopathogenic viruses and provides a variety of choices for the optimal MDV or a combination of multiple MDVs to target certain mosquitoes. During a recent outbreak of dengue virus in Guangdong Province, field-collected *A. albopictus* were received by our laboratory from the cities of Guangzhou and Shenzhen in Guangdong Province for the detection of arboviruses. Mosquito specimens collected were identified to species and *A. albopictus* adults catches were pooled (*n* = 20/pool, 13 female adult pools and 10 male adult pools) then virus detection was performed with DENV and MDVs RT-PCR/PCR assays. Further investigation showed the presence of MDVs in a certain female adult pool. Further investigation showed the presence of MDVs in certain populations. In this paper, we report the isolation and characterization of a novel MDV isolated from wild-caught adult *A. albopictus* in the city of Guangzhou in Guangdong Province, China. The virus was designated Aedes albopictus densovirus-7 (AalDV-7) after the vector from which it was isolated. We analyzed the pathogenicity of AalDV-7 to larvae of the major dengue and Zika virus vectors *A. albopictus* and *A. aegypti* and the potential Zika virus vector *Culex quinquefasciatus*. The infection rate for the host and the viral concentrations of AalDV-7 *in vivo* were also evaluated to explore its potential for vector control.

## Materials and Methods

### Ethics Statement

The research did not involve any regulated animals or human subjects.

### Mosquito Cell Maintenance and Mosquito Rearing

*Aedes albopictus* C6/36 cells (ATCC Cat# CRL-1660, RRID: CVCL_Z230) were grown at 28°C in Roswell Park Memorial Institute (RPMI) 1640 medium (Gibco BRL, United States) supplemented with 10% fetal bovine serum (FBS; Gibco BRL, United States). The *A. albopictus* Foshan strain and the *C. quinquefasciatus* Guangzhou strain used in this work were established in the laboratory in 1981 and 1993, respectively, and were obtained from Guangdong Provincial Center for Disease Control and prevention. *A. aegypti* were collected from Haikou in Hainan Province. Mosquitoes were reared at a temperature of 28°C and a 70–80% relative humidity with a 14 h/10 h light/dark cycle. Larvae were reared in pans and fed finely ground fish food mixed 1:1 with yeast powder. Adult mosquitos were kept in 30 cm^3^ cages and allowed access to a cotton wick soaked in 10% sucrose as a carbohydrate source. Adult females were allowed to bloodfeed on commercial defibrillated mice blood (Solarbio^®^ Life Science, Beijing, China) 3 and 4 days after eclosion. Every batch of mosquitoes was examined by conventional PCR to ensure that the experimental mosquitoes were free from MDVs (data not shown).

### Isolation and Purification of AalDV-7 Particles

To investigate densoviruses, we collected *A. albopictus* adults from Guangzhou. Mosquitoes caught in the field were sorted and mixed with phosphate-buffered saline (PBS), and part of the sample was used to detect MDV infection by traditional PCR methods as previously described (Wei et al., 2006). The pool of mosquitoes exposed to MDV was triturated in RPMI 1640 medium and centrifuged at 12,000 rpm in a microcentrifuge for 20 min at 4°C to remove the cellular debris. The supernatant filtered through a 0.22 μm syringe driven filter was added to the virus-free C6/36 cells and a pan containing 500 ml distilled water to infect 1st–2nd instar *A. albopictus* larvae (*n* = 200).

After 5 days of infection, MDV-infected C6/36 cells were harvested by using cell scrapers and lysed by freezing and thawing, whereas the MDV-infected larvae were homogenized using a motorized tissue homogenizer (Tiangen Biotech, Beijing, China) and then centrifuged at 12,000 × *g* for 30 min to remove the cellular debris. The supernatant was filtered with 0.22 μm filters and then centrifuged at 100,000 × *g* for 3 h at 4°C to pellet virion particles. The virion pellets were resuspended in PBS and purified by Cesium chloride gradient (1.2–1.5 g/ml) at 150,000 × *g* overnight at 4°C. The virion band removed from the gradient was mixed with PBS and centrifuged at 150,000 × *g* for 3 h. Finally, the viral particles were resuspended in PBS for DNA extraction and TEM.

### TEM (Transmission Electron Microscopy)

Purified viral particles were applied to glow-discharged carbon-coated grids and negatively stained with 2% (w/v) phosphotungstic acid. Electron micrographs were recorded using an FEI Tecnai G2 F20 electron microscope at nominal magnifications of 50000× to 100000×.

### RACE Analysis of the AalDV-7 Transcript

Total RNA was isolated from AalDV-7-infected C6/36 cells 5 days postinfection (d.p.i.) with TRIzol Reagent (Life Technologies, Carlsbad, CA, United States). 5′ RACE and 3′ RACE experiments were performed with the SMARTer RACE cDNA Amplification Kit (Takara Bio, Inc., Clontech, United States) according to the manufacturer’s protocol with NS1/NS2 and VP gene-specific primers and nest primers, respectively. The sequences of the primers used for RACE analysis are listed in Supplementary Table S1. The amplified fragments were gel-eluted, subcloned into the pLB vector (Tiangen Biotech, China) and confirmed by sequencing (Thermo Fisher Scientific, CA, United States). At least 5 different clones were sequenced per sample.

### Viral Genome Identification and DsRed Transducing Vector Construction

The total encapsidated AalDV-7 genomic DNA was extracted from pure virion particles using a Viral DNA Extraction Kit (Takara, Dalian, China). Purified viral DNA was then separated in a 1% agarose gel and visualized by ethidium bromide (EtBr). AalDV-7 DNA was blunt ended by incubation for 15 min at room temperature with 10 units of the Klenow fragment and then purified using the E.Z.N.A.^TM^ Cycle-Pure Kit (Omega Bio-Tek, GA, United States), cloned into the *Eco*RV site of the pKMV plasmid and transformed into *Stbl3* chemically competent *E. coli* (Thermo Fisher Scientific, United States). The plasmids were extracted from 10 positive clones, and the inserted viral DNA was confirmed by DNA sequencing. Briefly, due to the failure to subclone the entire AalDV-7 genome in one step, three clones were selected for digestion with restriction endonucleases and ligated together to build a full-length clone. As a result, the infectious plasmid clone pAalDV-7 containing the entire AalDV-7 genome was constructed. The pNS1-DsRed plasmid was constructed by inserting the red fluorescent protein (RFP) DsRed coding sequence between the *Nde*I and *Nsi*I sites of AalDV-7. As a result, DsRed was fused to the C-terminus of NS1, and the NS1-DsRed fusion protein was under the control of the NS1 promoter (pNS1 or p7). Then, the DsRed coding sequence was subcloned in the C terminus of the NS2 protein between the *Acl*I and *Nsi*I sites, and the pNS2-DsRed plasmid was constructed. The DsRed coding sequence was also inserted between the *Csp45*I and *Nsi*I sites of AalDV-7 to replace the partial VP protein while maintaining the nuclear localization signals (NLSs), resulting in the construction of the pVPNLS-DsRed plasmid (Figure 6A).

### C6/36 Cell Line Transfection

The supercoiled pNS1-DsRed, pNS2-DsRed, and pVPNLS-DsRed plasmids used for transfection were prepared using an Endo-free Plasmid Purification Kit (Omega, United States) according to the manufacturer’s protocol. When cells grew to a 60–70% confluent monolayer in 6-well cell culture plates (Corning costar, United States), Lipofectamine 2000 reagent (Thermo Fisher Scientific, United States) was used to mediate transfection according to the recommendations of the manufacturer. The transfected cells were examined at a wavelength of 557 nm to detect DsRed expression.

### Expression and Intracellular Localization of AalDV-7

C6/36 cells were transfected with the p7NS1-DsRed and p61-DsRed vectors. The expression of NS1-DsRed and NLS-DsRed in cells was monitored with a Nikon Eclipse TE2000-S inverted microscope (Nikon Corp., Tokyo, Japan) 24 h posttransfection (h.p.t.). Nuclei were defined on the basis of 4′,6-diamidino-2-phenylindole (DAPI) staining. The C6/36 cells transfected with DsRed expression vectors were fixed in 4% paraformaldehyde in PBS at room temperature for 15 min and stained with 0.5 μg/ml DAPI in PBS for 15 min at 37°C. After washing twice with PBS and water, coverslips were mounted onto glass slides. Fluorescent signals from the fusion proteins were observed under an inverted fluorescence microscope, and photographs were taken using a Nikon ACT-2U digital camera (Nikon Corp., Japan). Data were then processed and superimposed using Adobe in Adobe^®^ Photoshop^®^ software version 9.0 (Adobe Systems Inc., San Jose, CA, United States).

### Molecular Evolution Analyses

NS1 protein sequences of the DVs (Supplementary Table S2) were aligned using Clustal X software (RRID: SCR017055) (Larkin et al., 2007) version 1.81 with the program’s default parameters. The evolutionary history was inferred by using the Maximum Likelihood method based on the Le Gascuel 2008 model (Le and Gascuel, 2008). The bootstrap consensus tree inferred from 1000 replicates is taken to represent the evolutionary history of the taxa analyzed (Felsenstein, 1985). Initial tree for the heuristic search were obtained automatically by applying Neighbor-Join and BioNJ algorithms to a matrix of pairwise distances estimated using a JTT model, and then selecting the topology with superior log likelihood value. A discrete Gamma distribution was used to model evolutionary rate differences among sites [five categories (+G, parameter = 5.6594)]. Evolutionary analyses were conducted in MEGA7 (Kumar et al., 2016). Phylogenies were rooted using PPV and ADV.

### Antibody Production

Peptide synthesis and the generation of rabbit antisera against conserved MDV NS1 amino acid sequences (fragments DVKNKDKEPIERT-C) were performed by Convenience Biology Corporation (CBC, Changzhou, China). The peptides were conjugated to KLH, and complete Freund’s adjuvant was used to elicit an immune response. The VP gene was cloned into the pET-28a (+) vector and transformed into *DH5α* competent cells. The expression vector was verified by DNA sequencing and then transformed into *Arctic Express (DE3) RIL* competent cells (Stratagene, United States) for expression. Protein was purified and refolded according to the protocols in the His-Bind Purification Kit and the Protein Refolding Kit (Novagen, San Diego, CA, United States). Polyclonal antibodies against VP were produced in rabbits by Convenience Biology Corporation (CBC, Changzhou, China). Polyclonal antibodies against NS1 and VP were purified using an ImmunoPure IgG Purification Kit (Pierce, United States) according to the manufacturer’s protocols, and the antibody titers were measured using ELISA.

### Western Blot Analysis

Total protein was extracted from MDV-infected cells 5 d.p.i. and then analyzed by SDS-PAGE using 4% stacking and 10% resolving acrylamide gel with a constant voltage of 100 V. Then, the proteins were electrotransferred onto a polyvinylidene difluoride (PVDF) membrane (Millipore Corp., Bedford, MA, United States). Membranes were incubated in blocking buffer (5% skimmed milk in PBS-T buffer [PBS buffer with 0.05% Tween 20]) for 2 h at room temperature, followed by incubation with rabbit polyclonal antibodies against NS1 and VP at a dilution of 1:1000 in blocking buffer overnight at 4°C. Then, they were washed extensively in PBS-T buffer and incubated with goat anti-rabbit IgG antibodies conjugated with horseradish peroxidase (HRP, 1:5000 dilution, Santa Cruz Biotechnology, Inc., United States) for 2 h at room temperature. The blots were washed with PBS-T, and bands visualized using a Clarity^TM^ Western ECL Substrate Kit (Bio-Rad Co., CA, United States).

### Virus Production and Quantification

The pUCA and pUCP infectious clones containing the AaeDV and AalDV-3 genomes, respectively, were kindly provided by Prof. Jonathan Carlson and have been previously described in detail (Afanasiev et al., 1991). AaeDV and AalDV-3 were generated by transfecting the corresponding infection clones (pUCA and pUCP, respectively) into C6/36 cells using Lipofectamine 2000. At 5 d.p.i., the cells were harvested and lysed by three freeze-thaw cycles and then centrifuged for 10 min at 1,000 × *g*. The collected supernatants were served as the AaeDV and AalDV-3 stocks, respectively. Wt AalDV-7 was used to inoculate 75-cm^2^ cell culture flasks containing C6/36 cells at a confluency of 70–80%. At 5 d.p.i, AalDV-7 stocks were collected as described above.

Non-encapsidated DNA in the stocks was removed by digestion with RNase-free DNase I (Takara, China) at 37°C for 1 h. Then, the reactions were incubated at 65°C for 15 min to heat-inactivate DNase I. The total encapsidated genomic DNA was extracted using a MiniBEST Viral RNA/DNA Extraction Kit Ver. 5.0 (Takara, China). To quantify the AalDV-7 genome copy number, a standard curve was first constructed by making serial 10-fold dilutions of a linear plasmid at known concentrations. qPCR reactions were performed using SYBR^®^ Select Master Mix (Thermo Fisher Scientific, United States), and the procedures were carried out as described previously (Liu et al., 2017).

### MDV Detection in Larvae

To explore the infection rate of AalDV-7 in mosquito larvae, 1st–2nd and 3rd–4th instar larvae (*n* = 10 per group) of *A. albopictus*, *A. aegypti*, and *C. quinquefasciatus* were exposed to AalDV-7 by exposure to virus at a final concentration of 1.00 × 10^8^ geq/ml in 100 ml distilled water. After incubation for 24 h at 28°C, the larvae were washed three times with deionized water and then transferred to pans containing 500 ml distilled water. After 12 h, the larvae were again washed three times with deionized water, and then the DNA was isolated using a TIANcombi DNA Lyse & Det PCR Kit (Tiangen Biotech, China). PCR amplification was then performed using the Maxima Hot Start Green PCR Master Mix (Thermo Fisher Scientific) with gene-specific primers. The final wash water was used as a control. The genes for ribosomal protein 7 in *A. aegypti* (*Aae*rpS7, VectorBase: AAEL009496) and *A. albopictus* (*Aal*rpS7, GenBank: JN132168), and actin in *C. quinquefasciatus* (*Cqu*actin, GenBank: AB454447) were used as endogenous controls. To detect the influence of temperature on the infection rate of larvae to AalDV-7, three species of mosquito larvae were exposed to AalDV-7 and incubated at 20 and 32°C, respectively, and AalDV-7 positive larvae were confirmed by traditional PCR method as described above.

To detect the relative viral dose in the mosquito larvae, 1st–2nd and 3rd–4th instar larvae of *A. albopictus*, *A. Aegypti*, and *C. quinquefasciatus* (*n* = 10 per group) were exposed to AalDV-7 using the method described above. The total DNA was extracted, and the absolute quantification of MDV DNA was performed with qPCR following the procedure described above. The DNA concentration was measured with a NanoDrop spectrophotometer (Thermo Fisher Scientific, United States), and the ratio of viral gene copy number to total DNA concentration was used to estimate the relative viral dose in the mosquito larvae and normalize for differences in mosquito size.

For each treatment described above, three independent biological replicates were analyzed. All quantifications were performed using an Applied Biosystems 7500 Real-Time PCR cycler (ABI 7500) and QuantStudio^TM^ Real-Time Software v1.3 (Applied Biosystems, CA, United States). All of the primers used in this study are listed in Supplementary Table S1.

### Pathogenicity of AalDV-7

Newly hatched 1st instar larvae were exposed to AalDV-7 (*n* = 100 per group) at concentrations ranging from 1 × 10^8^ to 1 × 10^11^ geq/ml. The control group was exposed to virus-free C6/36 cell culture medium under identical conditions to those of the treatment groups. Then, the mosquito larvae were washed three times with deionized water and transferred into dechlorinated tap water and regularly fed after 24 h of infection. The traditional PCR was used to confirm the MDV free of the final wash water. Larval mortality was scored every 24 h until the emergence of the adults. The sublethal effects of the serially diluted viruses were also assessed by the measurement of different biological parameters including the development time of the larvae and pupae, the rate of pupation and their emergence. Each experiment was repeated three times.

### Influence of Post-exposure Temperature on the Bioactivity of AalDV-7

Sometimes, the biological efficacy of bioinsecticdes were more vulnerable to some environmental factors, especially the temperature. Thus, the LD_50_ of AalDV-7 to larvae were also tested at 20 and 32°C. The highest temperature level (32°C) was selected because test population was collected from Haikou and Foshan cities of China, which daytime mean temperature were just about 32°C from April to October, and nighttime mean temperatures were just about 20°C from November to March (China Meteorological Administration, 2019).

### The Influence of Storage Temperature on the Bioactivity of AalDV-7

The activity of bioinsecticdes also can be altered if not stored or transported properly. To examine the influence of possible temperature fluctuations during storing and transportation on the biological efficacy of AalDV-7, the AalDV-7 stocks were stored at four conditions (30°C for 180 days, 40°C for 1 month, 50°C for 10 days, and 60°C for 1 h), and then the LD_50_ of AalDV-7 on three species mosquito larvae were assessed according the method described above in the Section “Pathogenicity of AalDV-7.”

### Vertical Transmission Assays

First to second instar larvae of *A. albopictus*, *A. aegypti*, and *C. quinquefasciatus* were exposed to 10^9^ geq/ml, 10^10^ geq/ml, and 10^10^ geq/ml, respectively, and reared to adults. Finally, the AalDV-7-exposed females that survived were allowed to mate in a cage with AalDV-7-negative males, and then females of the parent generation (F_0_) were fed on a commercial defibrinated mice blood and were individually isolated and reared at 27°C for egg laying. After oviposition, females were tested by the AalDV-7 specific PCR to confirm infection status. F_1_ eggs from each AalDV-7-positive female were then surface-sterilized by soaking in 0.05% sodium hypochlorite for 5–10 min, then in 70% ethyl alcohol for 1 min (three times), and finally washed with distilled water three times (Beaty et al., 1980) and transferred to a new sterile plastic cups (230 ml, 7 cm diameter on top) to hatch, to avoid any potential contamination. Ten 4th instar larval progeny (F_1_) from each infected female were collected in one pool, and screened by the PCR for the presence of AalDV-7. Then the minimal filial infection rate (MFIR) was calculated as the minimum number of mosquitoes infected with AalDV-7 with 95% confidence intervals (95% CI).

### Statistical Analysis

Survival curves were calculated using the Kaplan–Meier test. The log-rank test was used to analyze the differences between survival curves. The median lethal dose (LD_50_) and the median lethal time (LT_50_) were determined by probit analysis. The LT_50_ and development time between different treatments were compared by one-way analysis of variance (ANOVA) followed by the least significant difference (LSD) test. Relative viral dose in different groups were analyzed by one-way ANOVA and the LSD test. The pupation and emergence rates of the different treatment groups were compared by chi-square test. SPSS software version 20.0 (SPSS Inc., Chicago, IL, United States) was used for data analysis.

## Results

### Isolation and Identification of AalDV-7

**FIGURE 1:**
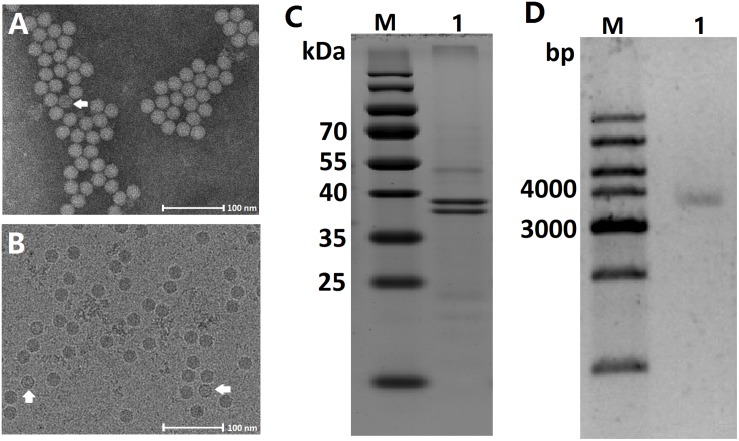
Characteristic of the AalDV-7 virion, capsid proteins, and genomic DNA. **(A)** Transmission electron micrograph of purified virions after negative staining. An empty particle is indicated by the white arrow. **(B)** Images of both full and empty AalDV-7 capsids (white arrows) embedded in vitreous ice can be observed. **(C)** Coomassie stained SDS-PAGE gel of purified AalDV-7 virions. Lane M: prestained protein ladder; Lane 1: purified AalDV-7 virions. **(D)** Agarose gel electrophoresis of AalDV-7 genomic DNA isolated from the purified AalDV-7 virion. Lane M: DNA ladder; Lane 1: dsDNA form of AalDV-7 genomic DNA.

Virions purified from both infected *A. albopictus* larvae and C6/36 cells shared characteristics typical of densoviruses as described previously and appeared as non-enveloped, isometric particles, 20 nm in diameter with perfect icosahedral symmetry when examined with negative stain (Figure 1A). Furthermore, the virions were embedded in amorphous ice and examined by cryo-electron microscopy. Both full and empty capsids were observed, and both types of capsid had similar smooth surfaces and sizes (Figure 1B). No obvious small and large particles were observed, indicating that viruses concentrated via ultracentrifugation were not contaminated with mosquito cellular debris.

The capsid proteins of purified virions were analyzed by SDS-PAGE, and Coomassie staining showed two bands corresponding to molecular masses of approximately 40 and 38 kDa (Figure 1C) representing two structural proteins, VP1 and VP2. VP2 was presumably derived from VP1 by proteolytic cleavage of a small peptide fragment from the amino terminus or alternatively by the internal initiation of translation at one of the downstream ATG codons (Afanasiev et al., 1999). To characterize the viral genome, the viral nucleic acids were separated by agarose gel electrophoresis and visualized by EtBr. The virus had a genome approximately 4 kb in size (Figure 1D).

### Viral Genome, Transcripts, and Protein Expression

**FIGURE 2:**
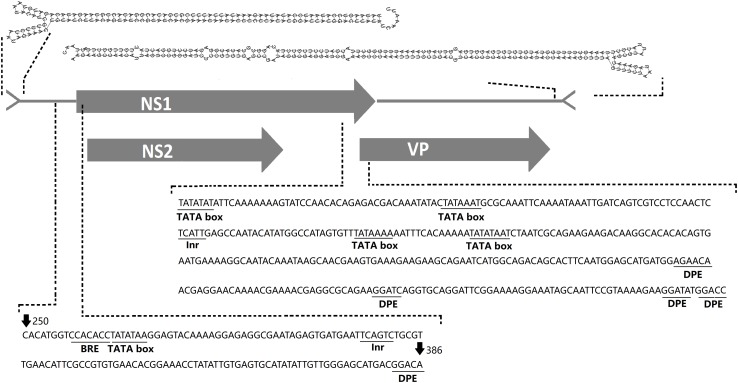
AalDV-7 genome organization. The gray rectangles indicate the three predicted overlapping ORFs in AalDV-7, NS1, NS2, and VP. Predicted 5-prime and 3-prime AalDV-7 terminal hairpin structures are shown at the top. Three core promoter elements (CPEs), the B recognition element (BRE), TATA box, and initiator element (Inr), and a downstream promoter element (DPE) are contained in the pNS1/NS2 and pVP promoter regions.

The complete genome sequence of the virus has been submitted to GenBank (GenBank: MK182384) and was termed AalDV-7. The viral genome is typical of MDVs. It is 4,048 nt in length, displays 98% nucleotide sequence identity with the Culex densovirus (GenBank: FJ805445, isolated from *Culex* mosquitoes collected in Xinjiang, China) and contains three overlapping ORFs, which is consistent with previous descriptions of other viruses within this genus. The 5′ ORF encoding the non-structural 1 (NS1) protein is 2,376 nt long, the middle ORF encoding the NS2 protein is 1,092 nt long and the 3′ ORF encoding the viral capsid protein (VP) is 1,071 nt long. The promoters for NS1 (pNS1 or p7) and NS2 (pNS2 or p7.4) are overlapping and are upstream of the initial codon for NS1, while VP is under the control of the pVP (p61) promoter (Figure 2). Three core promoter elements (CPEs) including the B recognition element (BRE), TATA box, and initiator element (Inr) and the downstream promoter element (DPE) are in the pNS1/NS2 and pVP promoter regions, whereas the MTE (motif ten element) was not found. The initial 146 bases at the 5′ end and the last 232 bases at the 3′ end of the genome consist of terminal inverted repeat sequences (IRs) and are predicted to fold into typical Y-shaped hairpin structures using RNAfold (Hofacker et al., 1994; Figure 2).

**FIGURE 3:**
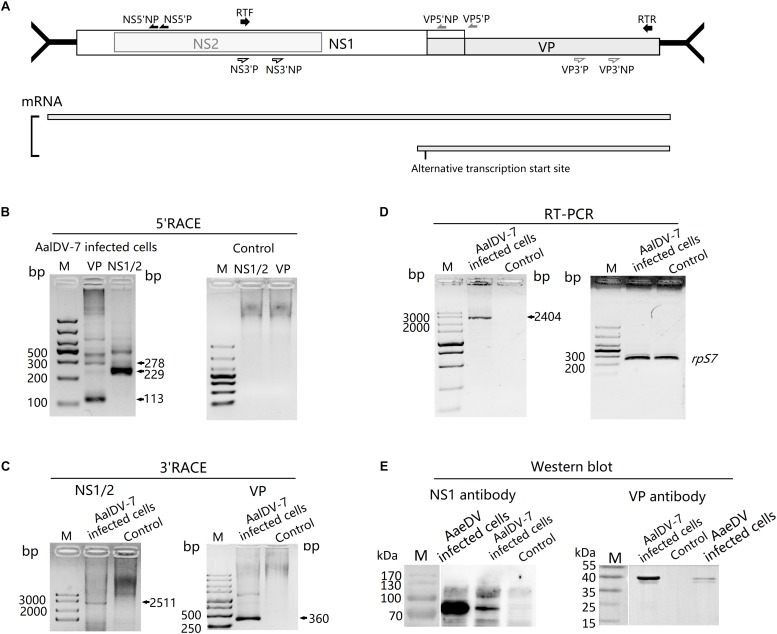
Transcript and protein expression analysis of AalDV-7. **(A)** Schematic representation of the AalDV-7 ORF, with arrows denoting the location of the primer sets used (upper panel). The transcript pattern of AalDV-7 mRNAs is indicated (lower panel). **(B)** 5′ RACE analysis of the AalDV-7 transcript. Total RNA was prepared from C6/36 cells exposed to AalDV-7 5 d.p.i. and control cells, and 5′ RACE was performed as described in the Section “Materials and Methods.” Lane M: DNA Ladder, **(C)** 3′ RACE analysis of the AalDV-7 transcript. **(D)** RT-PCR analysis of the AalDV-7 transcript. **(E)** Western blot analysis of AalDV-7. The AalDV-7-infected C6/36 cell lysate was analyzed by 10% SDS-PAGE and reacted with goat anti-rabbit IgG antibodies against the approximately 90 kDa non-structural protein and the 38/40 kDa structural protein. The mock-infected cell lysate was used as a negative control. The AaeDV-infected cell lysate was used as a positive control. Lane M, standard protein size marker.

To determine the transcription initiation site (TIS) for the two non-structural protein genes NS1 and NS2 and the structural protein gene VP, 5′ RACE was performed using RNA isolated from AalDV-7-infected C6/36 cells. The gene-specific primers were located at the NS1/NS2 common region and the VP gene region (Figure 3A). The results of the 5′ RACE experiments are shown in Figure 3B; for VP, two visible bands in the agarose gel 113 and 278 bp in size were specific products of the experiment, and the other faint visible bands in the gel were sequenced and confirmed to be non-specific amplification products. As a result, the TIS located 1 or 158 nt upstream of the ATG codon of VP indicated that the transcription initiation of the structural protein mRNAs occurs at an alternative site. Moreover, 5′ RACE analysis of the NS1/NS2 gene revealed two visible bands in the gel, and sequencing results showed that the 229 bp product was a specific product of the experiment, which revealed that the TIS is located 6 nt upstream of the ATG of the NS1/NS2 gene and that NS1 and NS2 gene share a common TIS. Transcription termination sites (TTSs) for the NS1, NS2, and VP genes were determined by 3′ RACE, as shown in Figure 3C. Only one strong band was detected following the VP 3′ RACE experiment, and DNA sequencing showed polyadenylation beginning 60 nt downstream of the termination codon for the VP gene. Furthermore, the 2511 bp NS1/NS2 3′ RACE PCR product revealed that the NS1/NS2 gene shared a common TTS with the VP gene. Since the NS1/NS2 and VP genes share a common transcription initiation site, it is theoretically possible that a 2,336 bp product would be obtained from VP 5′ RACE, but the absence of this product may have been caused by the long DNA templates and complicated secondary structure. Therefore, this transcript was reconfirmed by RT-PCR, and the results were consistent with expectation (Figure 3D).

To analyze AalDV-7 NS1 and VP protein expression, Western blots were performed on mock- and AalDV-7-infected C6/36 cells with polyclonal antibodies against the NS1 and VP proteins. A single band of approximately 90 kDa reacted with polyclonal antibodies against NS1 only in AalDV-7-infected C6/36 cells, while no visible band was detected in the control (Figure 3E). However, VP polyclonal antibodies reacted with two bands located at 40 and 38 kDa that corresponded to VP1 and VP2 (Figure 3E) present at the same positions as those shown in SDS-PAGE (Figure 1C).

### Molecular Phylogenetic Analysis

**FIGURE 4:**
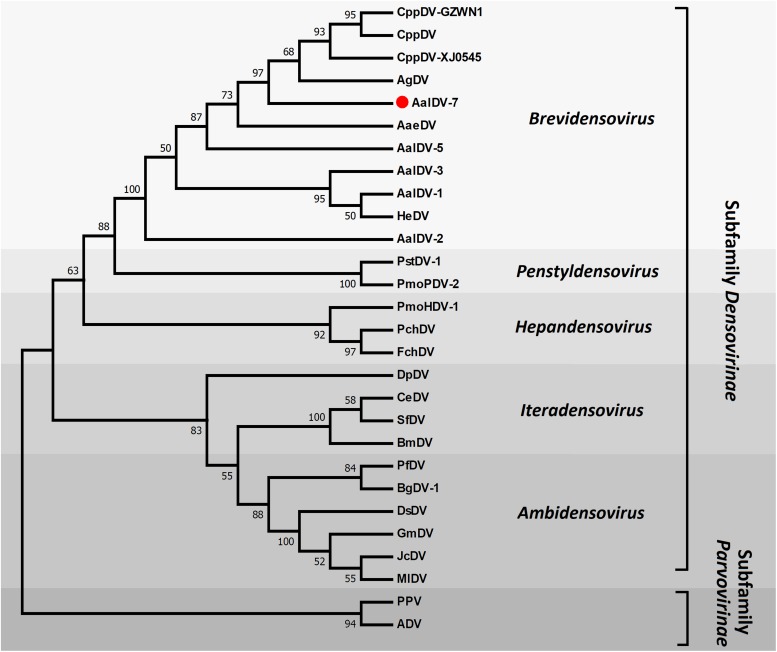
Molecular phylogenetic analysis of DVs NS1. The phylogenetic tree is based on a comparison of NS1 protein sequences. Numbers for interior nodes represent bootstrap and confidence probabilities based on 1000 replicates, followed by Maximum Likelihood method. The topology was rooted with PPV and ADV. Only bootstrap values greater than 50% are shown.

To determine the phylogenetic position, AalDV-7 was aligned with other densoviruses to reconstruct their evolutionary relationships. Only the NS1 gene product has motifs conserved among all parvoviruses, so the NS1 protein was used to align homologous sequences from other members of the *Densovirinae* subfamily of the *Parvoviridae* family representative of invertebrate and vertebrate densoviruses. We use PPV and ADV from the subfamily *Parvovirinae* as outgroups to root the tree. The topology obtained for the NS1 phylogeny showed high confidence levels in the defined groups (Figure 4). As expected, based on their phylogenetic relationships, all members of the *Densovirinae* subfamily were grouped into five clades, which was consistent with the present classification of the *Densovirinae* subfamily including five distinct genera, namely, *Ambidensovirus*, *Brevidensovirus*, *Hepandensovirus*, *Penstyldensovirus*, and *Iteradensovirus*. PPV and ADV clustered in a basal clade. MDVs were categorized into two clades (Sangdee and Pattanakitsakul, 2013). One clade consisted of AalDV-1, AalDV-3 and HeDV, and another clade consisted of AalDV-5, AalDV-7, AaeDV, CppDV, and AgDV, most of which were isolated from indigenous mosquitoes.

### Characteristics of the Three Putative Proteins of AalDV-7

**FIGURE 5:**
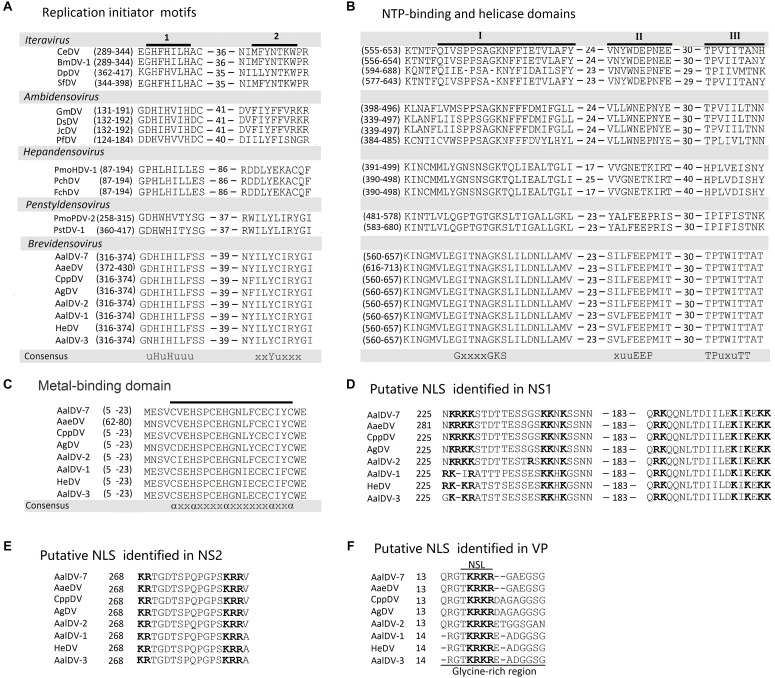
Comparison of the major domains in AalDV-7 proteins with those in the proteins of other DVs. **(A)** The location of two conserved motifs (uHuHuuu and xxYuxxx) in NS1 from AalDV-7 and other DVs. A u represents an hydrophobic amino acid, and x represents any residue. **(B)** The location of the NTP-binding domain (I) (GxxxxGKS) and putative helicase domains (II and III) (xuuEEP and TPuxuTT) in NS1 from AalDV-7 and other DVs. A u represents an hydrophobic amino acid, and x represents any residue. **(C)** The location of putative metal-binding domains in NS1 from AalDV-7 and other MDVs (axxaxxxxaxxxxxxaxxa) are underlined in each viral protein shown in the figure. The locations of the motifs are shown in brackets. An a represents a cysteine or histidine residue, and x represents any residue. The putative NLS motifs identified in the C-terminal regions of NS1, NS2, and VP from mosquito densoviruses. **(D)** NLS and bipartite NLS motifs in NS1. **(E)** NLS in NS2. **(F)** NLS in VP, with a glycine-rich region underlined. Basic NLS motifs are in boldface and separated by 10 aa in NS1 and 11 aa in NS2.

NS1 (or Rep) from members of the *Parvoviridae* family served as rolling circle replication (RCR) initiator proteins and directed the process of single-stranded viral DNA replication as following: binding to specific motifs within the viral ITRs, site- and strand-specific endonuclease (nicking) activity, and ATP-dependent helicase activity (Im and Muzyczka, 1992; Owens et al., 1993; Ward and Berns, 1995; Wonderling et al., 1995; Linden et al., 1996; Smith et al., 1999). Three conserved motifs in the RCR initiator protein family were associated with single-strand nicking activities (Chiorini et al., 1999), two of which were identified in AalDV-7 NS-1. These two RCR motifs are located in the amino terminal half of NS1; motif 1 is located at residues 316–374 and contains two consensus histidine residues, and motif 2 contains the active tyrosine involved in nicking activity (Nuesch et al., 1995; Walker et al., 1997; Figure 5A). An NTP-binding domain (NBD) and a putative helicase domain (HD) were located in the middle of NS1 (Figure 5B). Furthermore, a putative N-terminal metal-binding domain (MBD) that is a vital catalytic unit in NS1 endonuclease was observed in AalDV-7 as well as the MDVs (Berg, 1986; Tsonis et al., 1988; Miranda and Thompson, 2016; Figure 5C).

Nuclear localization signals (NLSs) are short peptide motifs that direct proteins to the nuclear pore complex and pass proteins through the nuclear membrane. The AalDV-7 NS1 protein was scanned using the WOLF PSORT program (Horton et al., 2007), and a potential NLS and several so-called bipartite NLSs were detected in the middle and C-terminal parts of the protein (Figure 5D). The pattern of this type of signal sequence was as follows: 2 basic amino acids and a 10 residues spacer followed by a region consisting of at least 3 basic residues out of 5 residues (Dingwall and Laskey, 1991). The NS2 ORF is embedded in the NS1 ORF (Figure 2). NS2 proteins in MDVs are involved in efficient DNA replication and the production of infectious progeny (Azarkh et al., 2008). A putative NLS was identified in the C-terminal region of the NS2 protein that is conserved among the MDVs (Figure 5E). An NLS-like domain was also found in the glycine-rich region (GRR) of the N-terminus of the AalDV-7 VPs, which is a conserved sequence among MDVs (Figure 5F). This GRR is common to the structural proteins of all the mammalian parvoviruses and is a substrate for proteolytic cleavage that generates the small capsid proteins observed in other parvoviruses (Cotmore and Tattersall, 1987). No typical conserved phospholipase A2 (PLA2) domain, which plays a key role in viral infectivity in other parvoviruses, was found in VP1 of the densoviruses from the genus *Brevidensovirus* (Zadori et al., 2001).

### Intracellular Localization of Capsid and Regulatory Proteins in Mosquito Cells

**FIGURE 6:**
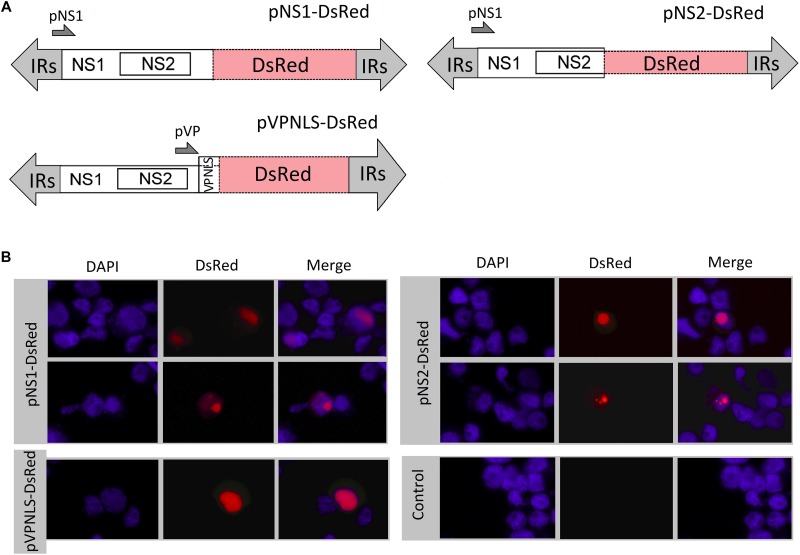
Subcellular localization of NS1, NS2 and VP in C6/36 cells. **(A)** Schematic organization of recombinant AalDV-7 plasmids. In plasmids p7NS1-DsRed, p7NS2-DsRed, and pVPNLS-DsRed, the DsRed gene were fused to the NS1, NS2 and VP gene, respectively. **(B)** The DsRed fusion protein distribution in cells was visualized, with red fluorescence showing the localization of the NS1, NS2 and VP proteins. Cells were counterstained with 4′,6-diamidino-2-phenylindole (DAPI) to show the nuclear localization (DAPI stains nuclei, blue fluorescence), and the merged field images aligned using Adobe Photoshop (merged, right panel). The three proteins were mainly uniformly distributed in the nucleus (upper panel for pNS1-DsRed and pNS2-DsRed), and NS1 and NS2 also displayed a punctate distribution in the nucleus (lower panel for pNS1-DsRed and pNS2-DsRed).

To accurately define the subcellular localization of AalDV-7-encoded proteins in mosquito cells, C6/36 cells were transfected with pNS1-DsRed, pNS2-DsRed, and pVP-DsRed, and the expressed DsRed proteins were visualized and photographed directly under fluorescence microscopy. DsRed expression was observed in pNS1-DsRed-transfected cells as early as 4 h.p.t., whereas DsRed expression was observed 8 h.p.t. in pNS2-DsRed-transfected cells and 12 h.p.t. in pVP-DsRed-transfected cells. The maximal fluorescent intensity was detected approximately 48 h.p.t. Both NS1 and NS2 DsRed fusion proteins were located in both the nucleus and cytoplasm of C6/36 cells but were located predominantly in the nucleus (Figure 6B). The three proteins were uniformly distributed in the nucleus (upper panel for pNS1-DsRed and pNS2-DsRed), and NS1 and NS2 also displayed a punctate distribution in the nucleus (lower panel for pNS1-DsRed and pNS2-DsRed). The NLSs of NS1, NS2, and VP indeed exerted their function, transporting proteins from the cytoplasm into the nucleus and ensuring that NS1 and NS2 functioned in viral DNA replication and that VP accumulated in the nucleus for viral assembly.

### Portal of Entry and Tissue Tropisms of AalDV-7 in Larvae

The larvae of *A. albopictus*, *A. aegypti*, and *C. quinquefasciatus* were exposed to mixed viral stocks, respectively. DsRed fluorescent protein as a marker was observed under the fluorescence microscopy. The newly emerging fluorescent larvae were singled out continuously from 1 to 3 d.p.i. The clearly visible DsRed positive larvae of *A. albopictus*, *A. aegypti*, and *C. quinquefasciatus* were 51.33 ± 6.03%, 55.83 ± 4.86%, and 40.25 ± 4.60%, respectively, and the initial visible fluorescent tissues which indicated the portal of entry of the virus were recorded, and percentage of different fluorescent tissue in total fluorescent larvae were calculated. As a result, 41.78 ± 2.96% and 40.24 ± 1.76% of *A. albopictus* and *A. aegypti* larvae showed initial fluorescence in more than one tissue site, whereas others shown an unique tissue location.

**FIGURE 7:**
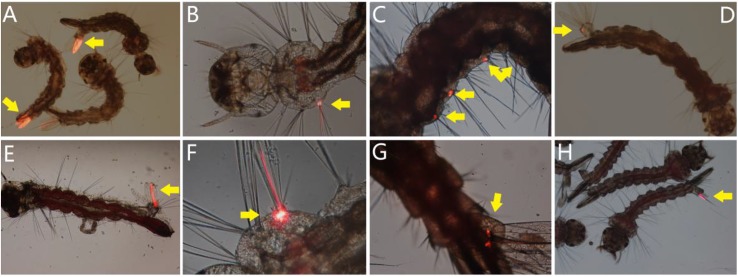
Portal of entry of AalDV-7 in mosquito larvae. The larvae were exposed to mixed viral stocks. DsRed fluorescent protein as a marker was observed, and the initial visible fluorescent tissues indicated the portal of entry of the virus. **(A)** Anal gills of *A. albopictus* larvae; **(B)** bristle cell of *A. albopictus* larva; **(C)** multiple bristle cells of *A. albopictus* larva; **(D)** base of anal gills of *A. albopictus* larva; **(E)** anal gills and bristle cell of *A. aegypti* larva; **(F)** the bristle cell of *A. aegypti*; **(G)** base of anal gills of *A. aegypti* larva; **(H)** anal gills of *C. quinquefasciatus* larvae.

The most frequent portal of entry were observed as anal gills (the sum of anal gills as unique tissue and one of multiple fluorescent tissues), accounting for 58.77 ± 4.60% (unique location 26.98 ± 4.75%) and 63.48 ± 3.41% (unique location 28.24 ± 3.06%) of *A. albopictus* and *A. aegypti* larvae were first showed red fluorescence in the anal gills. The bristle cell was detected as the second most common portal of virus entry, 51.09 ± 1.71% (unique location 20.65 ± 2.49%) and 53.88 ± 3.31% (unique location 22.45 ± 3.50%) of *A. albopictus* and *A. aegypti* larvae first appear fluorescence in bristle cells. The third high-frequency fluorescent tissue was the base of an anal gill in anal segment, which accounts for 22.69 ± 3.72% (unique location 7.05 ± 1.30%) and 22.42 ± 6.17% (unique location 7.00 ± 3.80%) of *A. albopictus* and *A. aegypti* larvae. However, for *C. quinquefasciatus*, only the anal gills were observed as the initial fluorescent site (Figure 7).

**FIGURE 8:**
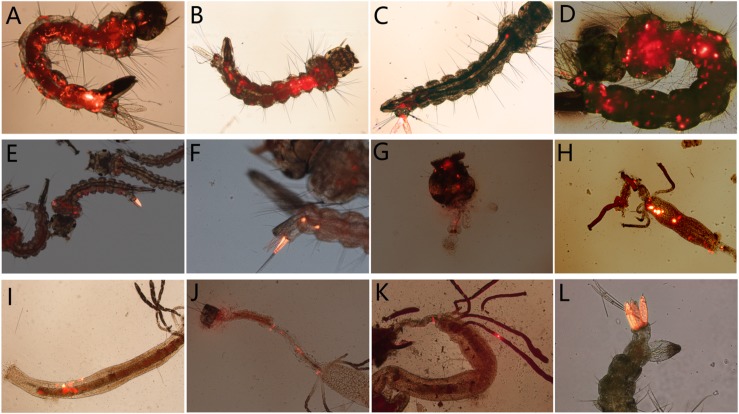
The distribution of AalDV-7 in mosquito larvae disseminated from the portal of entry; **(A,B)** *A. albopictus* larva whole body; **(C,D)** *A. aegypti* larva whole body; **(E)** *C. quinquefasciatus* larva whole body; **(F)** 8th segment of *C. quinquefasciatus* larva; **(G)** head of *A. albopictus* larva; **(H)** midgut of *A. aegypti* larva; **(I)** midgut of *A. albopictus* larva; **(J)** hindgut of *A. albopictus* larva; **(K)** Malpighian tubules of *A. albopictus* larva; **(L)** multi-anal papillae of *A. aegypti* larva.

Then the individually reared fluorescent larvae were monitored daily to detect the dissemination of virus in larval body within 5 days. As a result, in the 78.36 ± 1.46%, 75.74 ± 2.91%, and 75.29 ± 7.31% of *A. albopictus*, *A. aegypti*, and *C. quinquefasciatus* fluorescent larvae, Red fluorescence was observed spread to other tissues from its portal of entry, including muscle fibers, foregut, midgut, hindgut, malpighian tubule, etc. (Figures 8A–K); or disseminated from one anal gill to others (Figure 8L).

### The Pathogenesis of AalDV-7

**FIGURE 9:**
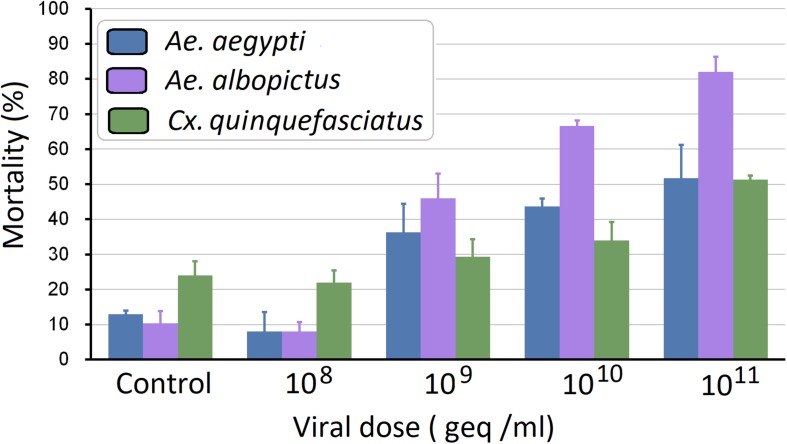
Mortality of *A. aegypti*, *A. albopictus*, and *C. quinquefasciatus* postinfection with AalDV-7. Newly hatched 1st instar *A. aegypti*, *A. Albopictus*, and *C. quinquefasciatus* larvae (*n* = 100 per group) were exposed to AalDV-7 at doses from 10^8^ to 10^11^ geq/ml. The bars indicate SDs from three independent (biological) replicates (*n* = 100 per replicate).

To investigate the pathogenesis of AalDV-7 in mosquitoes, 100 newly hatched 1st instar *A. albopictus*, *A. aegypti*, and *C. quinquefasciatus* larvae were exposed to serially diluted doses of AalDV-7 as described in the Section “Materials and Methods.” Viral stocks were quantitated by the qPCR assay. The pathogenesis of AalDV-7 in mosquito larvae was assessed by measuring the overall mortality. In general, when the viral dose reached 1 × 10^9^ geq/ml, the mortality of the *A. albopictus* and *A. aegypti*-infected larvae were significantly higher than that of the control group (*P* < 0.05), whereas a higher mortality of the infected *C. quinquefasciatus* larvae than that of the control was observed at a titer of 1 × 10^10^ geq/ml (*P* < 0.05). Then, mortality increased in the virus-infected groups in a dose-dependent manner (Figure 9). Furthermore, with viral doses from 1 × 10^9^ to 1 × 10^11^ geq/ml, AalDV-7-infected *A. albopictus* larvae always displayed a higher mortality rate than AalDV-7-infected *A. aegypti* and *C. quinquefasciatus* (*P* < 0.05). The LD_50_ with 95% CI was determined by probit analysis, and as shown in Table 1, the LD_50_ values of AalDV-7 against *A. albopictus*, *A. aegypti*, and *C. quinquefasciatus* were 10^9.48^ geq/ml (95% CI, 10^9.26^–10^9.69^ geq/ml), 10^10.56^ geq/ml (95% CI, 10^10.17^–10^11.15^), and 10^11.15^ geq/ml (95% CI, 10^10.58^–10^12.18^), respectively.

**TABLE 1:** LD_50_ of *A. aegypti*, *A. Albopictus*, and *C. quinquefasciatus* larvae exposed to AalDV-7.

**Mosquito**	**LD_50_ (geq/ml) (95% CI)**
***A. aegypti***	10^10.56^ (10^10.17^–10^11.15^)
***A. albopictus***	10^9.48^ (10^9.26^–10^9.69^)
***C. quinquefasciatus***	10^11.15^ (10^10.58^–10^12.18^)

**FIGURE 10:**
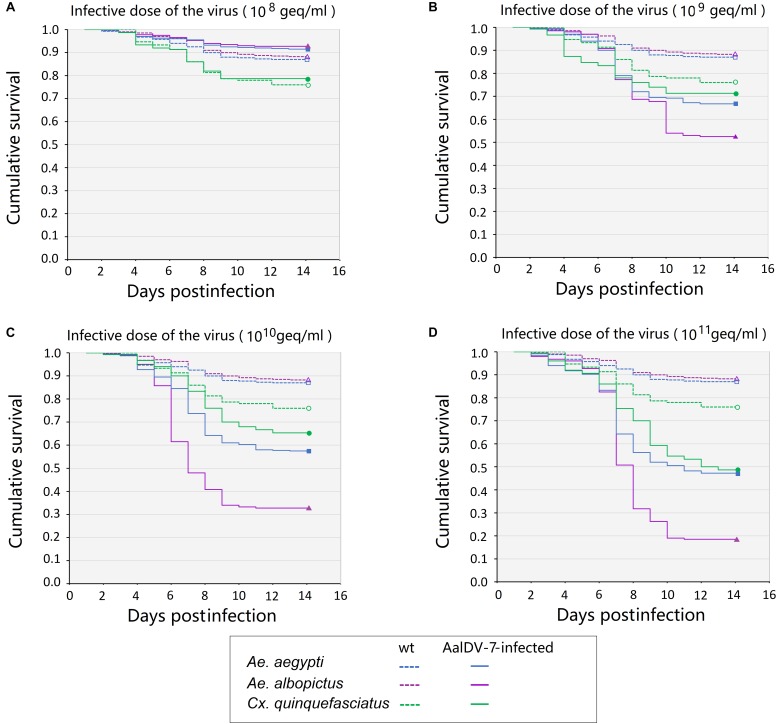
The survival curves for three mosquito species postinfection with serial AalDV-7 viral doses. The 1st instar larvae were exposed to AalDV-7 serial AalDV-7 viral doses. The data are presented as Kaplan–Meier survival curves from the three independent (biological) replicates (*n* = 100 per replicate). **(A)** Larvae survival curves viral doses of 10^8^ geq/ml, **(B)** 10^9^ geq/ml, **(C)** 10^10^ geq/ml, and **(D)** 10^11^ geq/ml.

Fourteen day Kaplan–Meier survival curves were constructed for *A. albopictus*, *A. aegypti*, and *C. quinquefasciatus* larvae exposed to viral doses of 10^8^ geq/ml, 10^9^ geq/ml, 10^10^ geq/ml, and 10^11^ geq/ml for 24 h, respectively. As shown in Figure 10, the survival of *A. albopictus* and *A. aegypti* exposed to 1 × 10^9^ geq/ml, 1 × 10^10^ geq/ml, and 1 × 10^11^ geq/ml, respectively, was significantly lower than that of the control groups (*P* < 0.05). *C. quinquefasciatus* only showed a significant reduction in longevity compared with that of the control groups at the high concentration of 1 × 10^11^ geq/ml (*P* < 0.05). Furthermore, at concentrations of 10^9^ geq/ml, 10^10^ geq/ml, and 10^11^ geq/ml, the lifespan of *A. albopictus* was significantly shorter than those of *A. aegypti* and *C. quinquefasciatus* (*P* < 0.05). Time-mortality data were subjected to probit analyses to estimate LT_50_ values of the three species of mosquito larvae exposed to 10^10^ geq/ml and 10^11^ geq/ml AalDV-7 (Table 2). Following infection with an AalDV-7 concentration of 10^11^ geq/ml, the LT_50_ values for *A. albopictus*, *A. aegypti*, and *C. quinquefasciatus* were 7.72 days (95% CI, 7.15–8.29 days), 10.24 days (95% CI, 9.67–10.91 days), and 10.42 days (95% CI, 9.98–10.91 days), respectively, and AalDV-7-infected *A. albopictus* display significantly lower LT_50_ values than AalDV-7-infected *A. aegypti* and *C. quinquefasciatus* (*P* < 0.05). However, following infection with an AalDV-7 concentration of 10^10^ geq/ml, the mortality of only AalDV-7-infected *A. albopictus* was more than 50%, and the LT_50_ was 9.49 days (95% CI, 8.91–10.11 days). The LD_50_ and LT_50_ values indicated that AalDV-7 was more pathogenic against *A. albopictus* than *A. aegypti* and *C. quinquefasciatus*.

**TABLE 2:** LT_50_ of *A. aegypti*, *A. Albopictus*, and *C. quinquefasciatus* larvae exposed to AalDV-7.

**Mosquito**	**LT_50_ (days) (95% CI)**	
	**10^10^ geq/ml**	**10^11^ geq/ml**
***A. aegypti***	N	10.24 (9.67–10.91) a
***A. albopictus***	9.49 (8.91–10.11)	7.72 (7.15–8.29) b
***C. quinquefasciatus***	N	10.42 (9.98–10.91) a

*N means viral dose lower than LD_50_. The LT_50_ values were calculated from three independent (biological) replicates (*n* = 100 per replicate). Means within the same column with different letters are significantly different (*p* ≤ 0.05).*

### The Sublethal Effects of AalDV-7

To assess the potential sublethal effects of AalDV-7, *A. albopictus*, *A. aegypti*, and *C. quinquefasciatus* larvae were exposed to AalDV-7 at concentrations under the LD_50_ of the virus (Table 1), the mean (±SD) time to pupation and emergence were recorded, and the percent pupation rate (PR, number of pupae per number of larvae × 100%) and percent emergence rate (ER, number of adults per number of pupae × 100%) were calculated (Supplementary Table S3). The three mosquito species did not exhibit delayed pupation and emergence when exposed to AalDV-7 at concentrations under the LD_50._ The PR and ER of *A. albopictus* and *A. aegypti* larvae exposed to AalDV-7 was decreased compared with those the control at viral doses of 10^9^ geq/ml (*P* < 0.05). The PR of the *C. quinquefasciatus* larvae decreased when exposed to 10^10^ geq/ml and 10^11^ geq/ml AalDV-7 (*P* < 0.05), and its ER decreased only after exposure to 10^11^ geq/ml AalDV-7 (*P* < 0.05).

### Infection Rate and Relative AalDV-7 Titer in Mosquitoes

**FIGURE 11:**
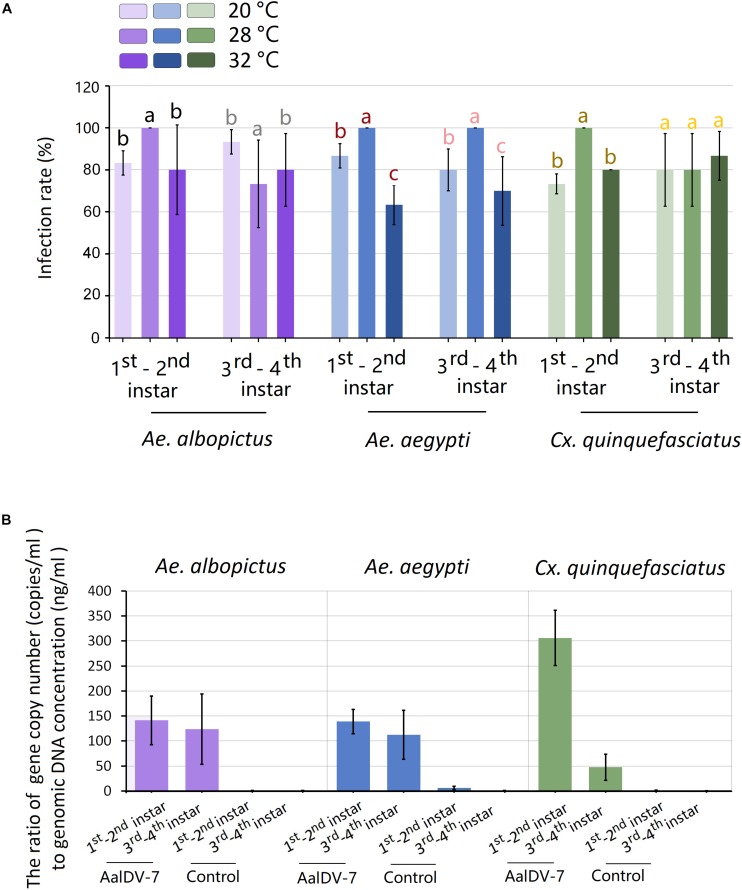
Infection rate and relative AalDV-7 titer in mosquito larvae. **(A)** Infection rates of *A. aegypti*, *A. albopictus*, and *C. quinquefasciatus* larvae when exposed to 10^8^ geq/ml AalDV-7 at 24 h.p.i. under different temperature. **(B)** The relative AalDV-7 titers in mosquito larvae, with the *Y*-axis representing the ratio of the viral gene copy number to the total DNA concentration (geq/ng). The error bars indicate SDs from three independent (biological) replicates (*n* = 10 per replicate). Different letters with the same color above bars represent significant differences in relative expression levels at the *P* < 0.05 level.

To explore whether the AalDV-7 infection rate in different mosquito species was significantly different maintained at the standard temperature (28°C), the traditional PCR method was used to confirm the infection rate of AalDV-7 in *A. albopictus*, *A. aegypti*, and *C. quinquefasciatus* larvae 24 h.p.i. at viral doses of 10^8^ geq/ml. Furthermore, to determine whether the AalDV-7 infection rate varied depending on the different developmental stage of the mosquito, the infection rates of 1st–2nd and 3rd–4th instar larvae were compared. All three mosquito species showed high susceptibility to AalDV-7, especially in the early stage 1st–2nd instar mosquito larvae, and infection rates were 100% in all three replicates. However, the older, late-stage 3rd–4th instar *A. albopictus* and *C. quinquefasciatus* larvae were more resistant to AalDV-7 than the 1st–2nd instar larvae, and their infection rates were 73.33 ± 20.82% and 80.00 ± 17.32%, respectively. However, for the 3rd–4th instar *A. aegypti* larvae, the infection rate remained as high as that measured in the early stage larvae (Figure 11A). The PCR results are shown in Supplementary Figure S1.

To detect whether the relative AalDV-7 titer in different mosquito species was significantly different, quantification of MDV DNA was performed with qPCR. The mosquito body size changes continually following larval development; therefore, to normalize for different body sizes, the relative AalDV-7 titer (geq/ng) in the larvae was determined by calculating the ratio of the viral genome copy number (geq/ml) to the larvae genomic DNA dose (ng/ml). As shown in Figure 11B, the highest relative titer (305.97 ± 67.57 geq/ng) was observed in 1st–2nd instar *C. quinquefasciatus* larvae, which was two times higher than that of *A. albopictus* larvae (141.31 ± 59.74 geq/ng) (*P* < 0.05) and 1st–2nd instar *A. aegypti* larvae (138.78 ± 30.09 geq/ng) (*P* < 0.05). However, the relative titer of 3rd–4th instar *C. quinquefasciatus* larvae (47.40 ± 32.05 geq/ng) was twice lower than of that of *A. albopictus* larvae (123.80 ± 86.29 geq/ng) (*P* < 0.05) and 3rd–4th instar *A. aegypti* larvae (112.20 ± 84.6 geq/ng) (*P* < 0.05). There were no statistically significant differences between the relative titers measured in *A. aegypti* and *A. albopictus* in both the 1st–2nd instar (*P* > 0.05) and the 3rd–4th instar larvae (*P* > 0.05) stages. The relative titer of the early stage, 1st–2nd instar *C. quinquefasciatus* larvae was six times higher than that of the 3rd–4th instar larvae (*P* < 0.05), but no significant difference was found between the 1st–2nd instar and 3rd–4th instar *A. aegypti* and *A. albopictus* larvae (*P* > 0.05).

### Influence of Temperature on the Infection Rate of Larvae to AalDV-7

To detect the influence of temperature on the infection rate of larvae to AalDV-7, three species of mosquito larvae were exposed to AalDV-7 and incubated at different constant temperatures (20 and 32°C), and infection rate were compared with mosquito that maintained at 28°C. As indicated in Figure 11A, for mosquitoes held at constant 20°C, the infection rate of 1st–2nd instar larvae of *A. albopictus*, *A. aegypti*, and *C. quinquefasciatus* were 83.33 ± 5.77%, 86.67 ± 5.77%, and 73.33 ± 17.32%, respectively. As the temperature increased to 32°C, the infection rate of 1st–2nd instar larvae of *A. albopictus*, *A. aegypti*, and *C. quinquefasciatus* were 80.00 ± 36.33%, 63.33 ± 9.43%, and 80.00 ± 0.00%, respectively. As a result, infection rates of 1st–2nd instar larvae both at 20 and 32°C showed significantly lower than that at 28°C (*P* < 0.05). In general, which indicated that 1st–2nd instar larvae became more resistant to infection with AalDV-7 when temperature shifted from optimum temperature 28°C. Whereas, there was no obvious changing regularity of 3rd–4th instar larvae infection rate with temperature variation observed in among three mosquito species (Figure 11A).

### Influence of Post-exposure Temperature on the Bioactivity of AalDV-7 to Mosquito Larvae

To explore the post-exposure temperature influence of AalDV-7, the LD_50_ of AalDV-7 in larvae of three mosquito species under different constant temperature conditions were determined. As shown in Supplementary Table S4, exposed to the same concentration of virus, when the temperature decreased from 28 to 20°C, the mortality rate decreased as the temperature fell, and the LD_50_ of AalDV-7 for *A. albopictus* and *A. aegypti* increased by 2.75, and 1.35-fold, respectively. In contrast, while the temperature increased from 28 to 32°C, the LD_50_ of AalDV-7 for *A. albopictus* and *A. aegypti* also increased by 2.40- and 2.04-fold. For *C. quinquefasciatus* Guangzhou strain, the higher temperature show more impact on larval survival, and more than half of larvae in control group died at 32°C. So we did not calculate LD_50_ of AalDV-7 to *C. quinquefasciatus* at 32°C, however, the LD_50_ was also increased by 2.45-fold at 28°C compared to that observed at 20°C.

### The Influence of Storage Temperature on the Bioactivity of AalDV-7 to Mosquito Larvae

The LD_50_ of AalDV-7 stock stored at 30°C for 180 days, 40°C for 1 month, 50°C for 10 days, and 60°C for 1 h were shown in Supplementary Table S5. The results indicated that AalDV-7 tolerate storage for long periods under a relatively high temperatures (30°C for 180 days and 40°C for 1 month), and LD_50_ of AalDV-7 only increased 1.74- and 3.47-fold compared to that at 28°C, respectively. In contrast, stored for short periods under higher temperatures (50°C for 10 days and 60°C for 1 h) had more obvious effect on bioactivity of AalDV-7, and LD_50_ was significantly increased 9.33- and 22.90-fold, respectively.

### Vertical Transmission

A total of 16, 25, and 14 pools consisting of 160, 250, and 140 F_1_ 4th instar larvae of *A. albopictus*, *A. aegypti*, and *C. quinquefasciatus* were tested with PCR, respectively. As a result, 8 of the 16 pools (*A. albopictus*), 20 of the 25 pools (*A. aegypti*), and 10 of the 14 pools (*C. quinquefasciatus*), were AalDV-7 positive (Supplementary Table S6), indicating that one or more of the F1 progeny in the pool were infected with AalDV-7. So, AalDV-7 MFIR of *A. albopictus*, *A. aegypti*, and *C. quinquefasciatus* were estimated as 5.00% (95% CI, 1.61–8.40%), 8.00% (95% CI, 4.63–11.37%), and 7.14 (95% CI, 2.86–11.42%), respectively.

## Discussion

Mosquito-borne viral diseases such as the dengue fever, Zika, West Nile encephalitis or meningitis, yellow fever, chikungunya, St. Louis encephalitis, and La Crosse encephalitis (LAC) viruses cause a significant fraction of the global infectious disease burden. In particular, dengue fever was listed as one of the top ten threats to global health in World Health Organization [WHO] (2019); indeed, nearly 390 million people worldwide are infected with DENV per year, resulting in one million cases of disease (Bhatt et al., 2013). Mosquito control remains the most effective strategy to prevent and reduce MBVD transmission. Conventional synthetic pesticides have represented the most common and widely used method for vector control and play a crucial role in preventing and reducing the risk of MBVDs. However, the negative impact on ecosystems and broadly emerging insecticide resistance (IR) pose a serious threat to the sustainable use of pesticides for MBD control in the future. Thus, novel, alternative mosquito control approaches are urgently needed.

Insect entomopathogenic viruses (IEVs) that can reduce insect populations or vector capacity and hence decrease MBVD transmission have been isolated in more than one thousand insect species and can be produced as eco-friendly biopesticides. With the widespread use of IEVs as insecticides in agriculture, the future of propagating viruses for mosquito control has been vividly shown. MDVs possess several promising properties; for example, MDVs exhibit a narrow host spectrum, are non-toxic to mammals and non-target arthropods, spread naturally among mosquito populations, are relatively stable and are constantly present in larval breeding bodies of water (Buchatsky, 1989; Vasil’Eva et al., 1990; Jousset et al., 1993; Faisst and Rommelaere, 2000; El-Far et al., 2004). These characteristics give MDVs the potential to be used as mosquito bioinsecticides.

Although nearly all MDVs exerted detectable pathological effects on mosquitoes, different viral strains varied greatly in their pathogenicity to different mosquito species, even different geographic strains of the same species (Jousset et al., 1993; Barreau et al., 1994). For example, as they are different viral strains, AalDV-1 infection caused a more than 90% mortality rate in 1st instar *A. aegypti* larvae (Jousset et al., 1993), whereas AaeDV infection caused a 75.1% mortality rate (Ledermann et al., 2004). However, the mortality rates of HeDV- and AalDV-3-infected *A. aegypti* were not significantly different than that of control mosquitoes (Ledermann et al., 2004). On the other hand, AThDV showed a significantly different pathogenicity to *A. aegypti* and *A. albopictus*, and infection with AThDV caused 51 and 82% mortality rates, respectively, in these mosquito species (Kittayapong et al., 1999). Moreover, AalDV-1 and AThDV displayed completely different LT_50_ values upon infection of the Rexville D, Chachoengsao, and Bangkok strains of *A. aegypti* (Hirunkanokpun et al., 2008). Thus, the varied pathogenicity of MDVs poses an obstacle in the wide application of MDVs for vector control. The construction and enrichment of MDV pools to provide a variety of choices for certain target mosquitoes has been proposed to solve this problem, and this would provide alternate or multiple application strategies to delay the onset of resistance.

In addition, low pathogenicity MDVs also show important practical application value. First, these viruses can be developed as delivery vectors. For example, AgDV infection had a minimal impact on the survival and transcriptome profile of its host, making it a prospective tool for functional mosquito gene research (Ren et al., 2008). Second, coinfection/superinfection with DENV and MDVs had a negative impact on DENV infection and replication *in vitro* and *in vivo* (Wei et al., 2006; Mosimann et al., 2011). Therefore, low or non-pathogenic symbiotic MDVs associated with *Aedes* could be manipulated to alter the mosquito’s ability to be infected with and transmit flaviviruses or to reduce mosquito fecundity or lifespan.

In our study, AalDV-7 isolated in Guangzhou showed significantly different pathogenicity in three species of mosquitoes. The LD_50_ of AalDV-7 in *A. albopictus* (10^9.48^ geq/ml) was 12 and 46 times lower than those in *A. aegypti* (10^10.56^ geq/ml) and *C. quinquefasciatus* (10^11.15^ geq/ml), and the LT_50_ in *A. albopictus* (7.72 days) was shortened by 25% and 26% compared with those in *A. aegypti* (10.24 days) and *C. quinquefasciatus* (10.42 days), at a titer of 10^11^ geq/ml. The lower LD_50_ and shorter LT_50_ values observed in *A. albopictus* indicated that AalDV-7 displayed higher pathogenicity against the *A. albopictus* Foshan strain than that against the *A. aegypti* Haikou strain and the *C. quinquefasciatus* Guangzhou strain. Analysis of the sublethal effects also showed that AalDV-7 infection significantly decreased the pupation and emergence rates. The 1st–2nd instar larvae of all three mosquito species showed a nearly 100% infection rate, and the highest relative vial titer (305.97 ± 67.57 geq/ng) was observed in the 1st–2nd instar larvae of *C. quinquefasciatus*.

In the field test, temperature has a prominent impact on insecticide effectiveness. Temperature affects different biological traits of insects such as larval survival, life-span, fertility, fecundity, etc. (Yang et al., 1994; Dreyer and Baumgärtner, 1996; Infante, 2010), even the susceptibility to virus (Westbrook et al., 2010; Ephantus and Alto, 2011; Buckner et al., 2015). In this study, whether temperature decreased from 28 to 20°C or increased to 32°C, the LD_50_ of AalDV-7 for *A. albopictus* and *A. aegypti* increased. There were no positive or negative coefficient were observed between temperature and LD_50_ of AalDV-7. However, infection rates of *A. albopictus* and *A. aegypti* 1st–2nd instar larvae at 20 and 32°C showed significantly lower than that at 28°C, these results indicated the varied infection rate under different temperature maybe were the key factors that lead to different LD_50_.

Very little information regarding the effect of unmoral storage or transportation conditions on activity of MDVs, and these studies of bioinsecticides are limiting factors determining their performance. In current study, storage for long periods under a relatively high temperatures had only a limited impact on activity of AalDV-7. However, the storage for short periods under higher temperatures displayed the relative obvious influence. Therefore, the results indicated that conditions of storage or transportation studies should be taken into consideration when bioinsecticide is registered in future.

Several MDVs were confirmed can be vertically transmitted (Barreau et al., 1997; Kittayapong et al., 1999; Ren et al., 2008; Altinli et al., 2018), which not only results in relaxed selection pressure but also as a bonus, leading to MDVs spread into the mosquito population and increased coverage and efficacy (Ren and Rasgon, 2010). Our results showed similar results to the study previously described, revealing that high vertical transmission rate among all the three mosquito species, even exposed to AalDV-7 at the concentration under LD_50_ which indicated AalDV-7 can persist in mosquito populations and had pathogenic impacts on larvae continuously.

## Conclusion

In conclusion, these pathogenic characteristics indicate the potential of AalDV-7 as a mosquito control agent in China, whereas the negligible pathogenicity and higher infection rate and viral dose observed *in vivo* make it a candidate gene delivery vector in *C. quinquefasciatus*. Furthermore, the continuous discovery and isolation of new MDVs will enrich the pool of mosquito entomopathogenic viruses and provide a variety of choices for single or multiple combinations of MDVs to optimally target certain mosquitoes.

## Data Availability

The raw data supporting the conclusions of this manuscript will be made available by the authors, without undue reservation, to any qualified researcher.

## Author Contributions

JG conceived of the idea and designed the study. JL, YD, YS, ZL, and YZ conducted all the experiments. PL, YG, JG, and XC analyzed the data. JG wrote the manuscript and prepared all the tables and figures. XC reviewed the manuscript. All authors approved the manuscript.

## Conflict of Interest Statement

The authors declare that the research was conducted in the absence of any commercial or financial relationships that could be construed as a potential conflict of interest.
