# Supplementary material for: A Novel Densovirus Isolated From the Asian Tiger Mosquito Displays Varied Pathogenicity Depending on Its Host Species
Source: Front Microbiol. 2019 Jul 5;10:1549. doi: 10.3389/fmicb.2019.01549 (PMC6624781; doi:10.3389/fmicb.2019.01549)
Supplement: Supplementary file 1 [file Table_1.DOCX]

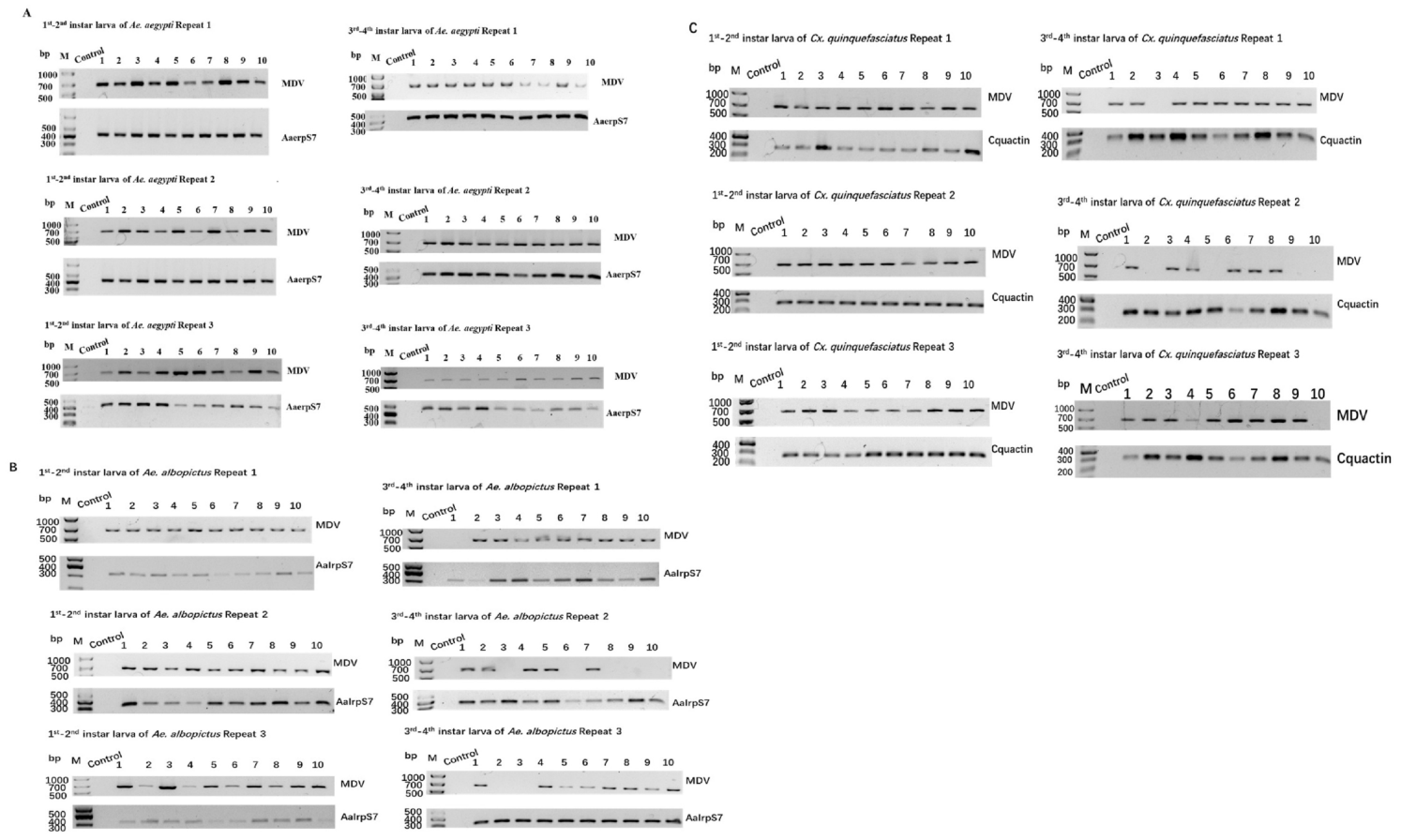


**Supplementary Figure 1.** **PCR results indicating infection rate of AalDV-7 in** 1**^st^-2^nd^ and 3^rd^-4^th^ instar larvae *of Ae. albopictus*, *Ae. aegypti* and *Cx. quinquefasciatus*.** LaneM: DNA ladder. Lane Control: The final water was used as a control. Lane 1-10: 10 mosquito larvae exposed to AalDV-7. (A) PCR results indicating infection rate of AalDV-7 in 1^st^-2^nd^ and 3^rd^-4^th^ instar larvae *of* *Ae. aegypti* and. (B)PCR results indicating infection rate of AalDV-7 in 1^st^-2^nd^ and 3^rd^-4^th^ instar larvae *of Ae. albopictus*. (C)PCR results indicating infection rate of AalDV-7 in 1^st^-2^nd^ and 3^rd^-4^th^ instar larvae *of Cx. quinquefasciatus*. The infection rates were calculated from three independent (biological) replicates (n=10 per replicate).
